# A Fair Share of Work: Is Fairness of Task Distribution a Mediator Between Transformational Leadership and Follower Emotional Exhaustion?

**DOI:** 10.3389/fpsyg.2019.02690

**Published:** 2019-11-29

**Authors:** Tabea E. Scheel, Kathleen Otto, Tim Vahle-Hinz, Torsten Holstad, Thomas Rigotti

**Affiliations:** ^1^International Institute of Management and Economic Education, Work and Organizational Psychology, Europa-Universitaet Flensburg, Flensburg, Germany; ^2^Department of Psychology, Work and Organizational Psychology, University of Marburg, Marburg, Germany; ^3^Department of Organizational, Business, and Social Psychology, Psychologische Hochschule Berlin, Berlin, Germany; ^4^DB Cargo AG, Mainz, Germany; ^5^Psychological Institute, Work, Organizational and Business Psychology, University of Mainz, Mainz, Germany

**Keywords:** transformational leadership, fairness of task distribution, follower well-being, emotional exhaustion, German employees

## Abstract

Drawing on social resource theory and the norm of equity, this research proposes fairness of task distribution as a mediating mechanism of the well-established relationship between transformational leadership and followers’ well-being, conceptualizing the latter as low emotional exhaustion. Using data from 479 German employees in a three-wave longitudinal study, we found transformational leadership to be related to fair task distribution over time. The perceived fairness of task distribution mediated the relationship between transformational leadership and follower emotional exhaustion (structural equation modeling) when excluding stabilities. Our results also show a reverse causation effect for emotional exhaustion and transformational leadership over a longer time period (within 20 months), suggesting a circular process, as well as a mediation by fairness of task distribution when excluding stabilities. The findings emphasize the importance of fair task distribution by leaders for followers’ well-being.

## Introduction

We aim to investigate how leaders’ ability to distribute tasks fairly (hereafter referred to as fairness of task distribution) in a team setting can potentially decrease followers’ emotional exhaustion. We specifically hypothesize that fairness of team task distribution, a common process in everyday working life and that is vital to team effectiveness (Mikula, 2002), mediates between transformational leadership and followers’ well-being. Task allocation (delegating), for which leaders are responsible (Van Knippenberg et al., 2007), is highly relevant to how and to what degree followers perceive leaders as fair (Sheppard and Lewicki, 1987). Fair task distribution has previously only been investigated in the context of household labor (Mederer, 1993; Mikula, 2002) or with reference to its specific effect on performance (Lipponen and Wisse, 2010). Given the importance of fairness to health outcomes (Elovainio et al., 2006), this is surprising.

Our study contributes to the existing studies on mediating mechanisms that links transformational leadership to (un)favorable outcomes, and to research in the fields of equity theory and social resource theory in its focus on fair task distribution as an important aspect with concrete implications for leadership development. This is the first study focusing on fairness of task distribution as mediator between transformational leadership and employee outcomes, and thus one of the rare studies to focus on a distribution of an unpopular rather than a positive allocation object.

Following a brief overview of transformational leadership and its relation to follower emotional exhaustion, we focus on fairness of task distribution and its mediating role between transformational leadership and emotional exhaustion.

### Transformational Leadership and Emotional Exhaustion of Followers

Leadership behavior has been identified as an important source of follower well-being (e.g., Van Dierendonck et al., 2004). Transformational leadership, a concept introduced by Burns (1978), includes inspiration, intellectual stimulation and individual consideration (e.g., Bass, 1999). Transformational leaders motivate their followers to stand together and to strive for a collective goal, stressing an important purpose (Bass and Riggio, 2006) and giving followers a sense that their work has a higher meaning (Nielsen et al., 2008). Transformational leadership and indicators of follower well-being are positively related (see review by Skakon et al., 2010). More importantly, significant negative associations between transformational leadership and employee’s emotional exhaustion have been found both cross-sectionally (Syrek et al., 2013) and longitudinally (Gregersen et al., 2014). Emotional exhaustion involves “feelings of being […] depleted of one’s emotional and physical resources” (Maslach et al., 2001, p. 399) and thus represents the dimension of burnout related to strain on the individual. Transformational leadership is related to health outcomes via different paths. Underlying mechanisms proposed include a leader’s ability to foster followers’ positive emotions (Bono and Ilies, 2006), followers’ own trust in leaders (e.g., Liu et al., 2010), and the characteristics of the work involved (Nielsen et al., 2008). In this study, we explore whether leaders’ fairness with respect to the distribution of tasks in a working team also functions as a mediator.

### The Mediating Role of a Fair Task Distribution

Leaders’ behavior greatly influences the perception of fairness on the part of the followers (Colquitt and Greenberg, 2003), since employees see fairness toward their subordinates as a key responsibility of leaders (Naumann and Bennett, 2000). Transformational leadership and fairness (i.e., procedural, interactional, distributive justice) are significantly positively related (Bacha and Walker, 2013; Gillet et al., 2013), and transformational leadership also relates to fairness at the team level (Cho and Dansereau, 2010). Transformational leaders provide individual support to each team member and challenge them in accordance with each persons’ resources. Thus, this type of leadership likely both increases team members’ general perceptions of fairness, and also positively influences the distribution of tasks within a team.

According to social resource theory (Foa, 1971; Foa and Foa, 1974), tangible goods (money, goods, services) and intangible ones (love, status, information) are exchanged via social relations. We propose task allocation to be yet another intangible resource that can be distributed within work teams. Fairness principles differ depending on whether a resource carries material or immaterial (symbolic) benefits (e.g., Sabbagh et al., 1994). Furthermore, the social context in which a distribution occurs is a prominent determinant of whether or not an allocation is perceived as fair (e.g., Deutsch, 1975). Given its status as an intangible resource and the fact that it refers to work context, fairness of task allocation should be perceived in accordance with the norm of equity (Deutsch, 1975); that is, a fair approach distributes tasks based on individual ability, contribution and effort (Sabbagh et al., 1994). Equality and need, as other criteria for fairness perception of outcome distribution (Colquitt et al., 2001), are less relevant in work relationships.

According to equity theory, employees expect a compensation that is in line with their contributions (Deutsch, 1975). What employees see as equitable depends on how they compare their own outputs and inputs vis-a-vis their co-workers, that is, on social comparisons (Festinger, 1954).

Moreover, according to Lerner (1987), the foundation of all social justice perceptions is the experience of entitlement: people judge that someone “is entitled to a particular set of outcomes by virtue of who they are or what they have done” (Lerner, 1987, p. 108). The distribution of outcomes in relation to a person’s actions is an important issue in exchange relationships, as supervisor-employee dyads are (Mikula, 2002). Employees contribute to the team with the expectation that they will receive a comparable contribution in return. Given their expectations that contributions should be allocated in proportion to these inputs (Mikula, 2002) and in relation to other’s inputs (Lerner, 1987), employees closely monitor individual inputs. Activities in work contexts are subject to “elaborate rules of entitlement and obligation” (Lerner, 1987, p. 109), which applies to work tasks. Individual consideration, as one behavior of transformational leaders, includes paying attention to employees (Nandedkar and Brown, 2018) and should include the ability to effectively know and address individual employees’ expectations of perceived entitlements with regard to work tasks. In this case, team members would perceive task distribution as fair, that is, as based on the principles of equity on the part of employees. Through their idealized influence and integrity, transformational leaders who act according to their values and ethical standards, are also perceived as trustworthy and fair (Nandedkar and Brown, 2018). We assume that transformational leaders will promote employees’ perception that a specific task distribution is fair if they are clear about different roles in their teams, set transparent goals, emphasize the importance of each individual contribution, highlight the idea of a common mission, and communicate the responsibilities of all team members.

The perception of violated entitlements based on social comparison (Festinger, 1954) provokes emotional reactions like frustration and disappointment (Lerner, 1987). Likewise, fairness is related to health outcomes such as reduced anxiety and depression (Spell and Arnold, 2007) and lowered stress levels (Cropanzano and Wright, 2010). By focusing on task distribution, our study addresses the most prevalent leadership task, that is, division of labor in a team to fulfill joint goals. Generally, distributive justice is defined as the fairness of outcome distributions and allocations (Cropanzano et al., 2001). The distribution of tasks differs from the distribution of outcomes (e.g., promotions) in that outcomes are usually valued resources, whereas tasks may be either preferred or not (Mikula, 2002). In a social comparison process (Festinger, 1954), employees might perceive the number of preferred tasks assigned to them as either greater or fewer than those of their team mates (Lipponen and Wisse, 2010), leading to unfavorable or favorable fairness judgments. Tasks can be assigned in accordance with criteria such as seniority and skills, or they can be undertaken by the whole team, or the responsibility for a specific task can rotate among team members. Task distribution can be organized in accordance with criteria such as the effectiveness or efficiency of task accomplishment, but fairness may also be considered (Mikula, 2002). Most likely, the norm of equity will be applied when the allocation of tasks in a team is in accordance with the individual contributions of team members, prompting employees either to perceive the allocation as fair, or to experience equity distress. If the equity norm is frequently perceived as violated, stress states may culminate in emotional exhaustion. For this reason, fairness has been shown to influence employee well-being and health (Elovainio et al., 2006), and fairness of task distribution has been related to lower emotional exhaustion in a cross-sectional study (QPSNordic, Lindström et al., 2000). In line with social comparison theory and equity principles, we propose that fair task distribution is negatively related to followers’ levels of emotional exhaustion over time.

On a general level, the mediating role of organizational justice dimensions has been demonstrated for the relationship between transformational leadership and criteria such as psychological health (Walsh et al., 2014), organizational citizenship behavior (OCB; Carter et al., 2014), and quality of work life (Gillet et al., 2013), organizational growth (Katou, 2015) and emotional exhaustion (Holstad et al., 2013). The dimension distributive justice was shown to mediate cross-sectionally between transformational leadership and quality of work life (Gillet et al., 2013) as well as between transformational leadership and (proposedly) positive work outcomes (e.g., OCB, task performance; Nandedkar and Brown, 2018). However, despite the fact that task distribution is a core leadership responsibility, to the best of our knowledge no studies to date have analyzed fairness of task distribution as a mediator between transformational leadership and employee outcomes.

In sum, a fair distribution of tasks may give transformational leaders an additional tool through which to promote follower well-being.

*H1*: Fair task distribution mediates the negative relationship between transformational leadership and follower emotional exhaustion.

## Materials and Methods

This study used a German subsample of a larger international research project (Rigotti et al., 2014). Employees of eight organizations (70.7% finance, 18.5% public administration, 4.8% education, 5.0% mechanical engineering) participated voluntarily and confidentially. Works councils approved of the study.

Three questionnaires with time lags were provided (lag T1-T2 13 months; T2-T3 7 months). The majority used an online survey (Unipark), but 10% filled in by paper-pencil-method. In order to match the responses to the different teams involved, each team was given a unique code. The latter was based *a priori* on team email lists provided by the organizations. Team members were given the same code.

Of the 1,336 German employees who responded at T1, 936 participated at T2 (70% response rate), and 724 responded at T2 and T3 (54% response rate). We excluded participants who did not provide data on all three measurement occasions and had a change in leadership within the measurement occasions. Of the remaining sample (see also Table 1) of 479 employees, 93 (21.1%) were men and 386 (78.9%) women. As is typical for service-oriented occupations, the share of women in our sample was larger than that of men. The mean age of respondents was 41.25 years (*SD* = 19.43), and mean team-tenure was 7.08 years (*SD* = 6.33). The attrition analysis for the main study variables showed that the longitudinal sample did not differ from the dropouts.

**TABLE 1:** Means, standard deviations, reliabilities and zero-order correlations between the study variables.

**Variable**	***M***	***SD***	**1.**	**2.**	**3.**	**4.**	**5.**	**6.**	**7.**	**8.**	**9.**	**10.**	**11.**	**12.**
1. Sex	0.19	0.40	–											
2. Age	41.25	9.43	−0.10^∗^	–										
3. Team tenure	7.08	6.33	–0.07	0.45^∗∗^	–									
4. Transformational leadership T1	3.22	0.88	–0.04	0.08	–0.00	(.94)								
5. Fair task distribution T1	3.59	0.99	–0.01	0.02	0.03	0.52^∗∗^	–							
6. Emotional exhaustion T1	2.49	1.44	–0.03	0.05	0.02	–0.22^∗∗^	–0.23^∗∗^	(.88)						
7. Transformational leadership T2	3.17	0.88	–0.05	–0.07	0.05	0.78^∗∗^	0.50^∗∗^	–0.15^∗∗^	(.94)					
8. Fair task distribution T2	3.56	0.97	0.04	0.05	0.12^∗^	0.45^∗∗^	0.43^∗∗^	–0.13^∗∗^	0.60^∗∗^	–				
9. Emotional exhaustion T2	2.57	1.44	–0.03	0.02	–0.05	–0.19^∗∗^	–0.17^∗∗^	0.69^∗∗^	–0.23^∗∗^	–0.24^∗∗^	(.87)			
10. Transformational leadership T3	3.14	0.88	–0.06	–0.06	–0.02	0.71^∗∗^	0.43^∗∗^	–0.18^∗∗^	0.81^∗∗^	0.49^∗∗^	–0.22^∗∗^	(.95)		
11. Fair task distribution T3	3.43	0.99	–0.00	0.04	0.06	0.39^∗∗^	0.46^∗∗^	−0.11^∗^	0.47^∗∗^	0.50^∗∗^	–0.14^∗∗^	0.57^∗∗^	–	
12. Emotional exhaustion T3	2.55	1.40	–0.01	0.03	0.06	–0.19^∗∗^	–0.17^∗∗^	0.65^∗∗^	–0.22^∗∗^	–0.21^∗∗^	0.73^∗∗^	–0.24^∗∗^	–0.17^∗∗^	(.87)

*^∗∗^*p* < 0.01, ^∗^*p* < 0.05, *N* = 429–479, Cronbach’s α in Parentheses.*

### Measures

The concept of transformational leadership has often been criticized for its lack of conceptual distinctiveness (Carless, 1998; Van Knippenberg and Sitkin, 2013). Most importantly, prior research (Carless, 1998; Kanste et al., 2007) has failed to reproduce the four-dimensional structure proposed by Bass and Avolio (1995). In line with previous research (Nielsen et al., 2008), we therefore measured transformational leadership as a one-dimensional construct, assessing *Transformational leadership* using the 7-item Global Transformational Leadership Scale developed by Carless et al. (2000) on a Likert scale ranging from 1 (*to a very small extent*) to 5 (*to a very large extent*). Cronbach’s α was .94 (T1; T2) and .95 (T3).

*Fairness of task distribution* was assessed with the item “Does your immediate superior distribute the work fairly and impartially?” from the general Nordic Questionnaire (QPSNordic; Dallner et al., 2000) with a 5-point Likert scale ranging from 1 (*very seldom or never*) to 5 (*very often or always*). The QPSNordic is an established scale (e.g., used as basic for the Copenhagen Psychosocial Questionnaire/COPSOQ, Kristensen et al., 2005). Also, single item measures were evaluated as being acceptably reliable and valid before (e.g., Fisher et al., 2016; for stress symptoms/QPSNordic, Elo et al., 2003).

*Emotional exhaustion* was measured with three prototypical items of the Maslach Burnout Inventory (MBI-GS, Maslach et al., 1996) on a scale from 1 (*never*) to 7 (*every day*). Cronbach’s α was .88 (T1) and .87 (T2; T3).

As age and gender are related to well-being (e.g., Mäkikangas and Kinnunen, 2003), both were included as *control variables*. Team tenure was controlled for, given that the hypothesized effects of leadership characteristics on subordinate well-being may take some time to become manifest in followers’ well-being.

### Statistical Analysis

We used Mplus 8 and the robust maximum likelihood estimator (MLR) to specify structural equation models (SEM) testing the proposed relationships in a cross-lagged panel design. For model comparisons we relied on common thresholds for fit indices (CFI/TLI values of >0.90, 0.06 for RMSEA, 0.08 for SRMR; Hu and Bentler, 1999). As χ2 statistics are sensitive to sample size, a value between 1 and 3 for a standardized χ2 divided by the degrees of freedom (χ2/df) has been proposed as acceptable (Wheaton et al., 1977; Moosbrugger and Schermelleh-Engel, 2007). In the current sample, employees were nested in work units. Therefore, we controlled for variance in group membership using the type complex command.

Measurement invariance over time is a precondition for investigating longitudinal relationships (Little et al., 2007). Accordingly, we tested measurement invariance for our measures of transformational leadership and emotional exhaustion, by comparing the model fit of a baseline model (identical measurement models on all three measurement occasions) against a weak invariance model (constraining the factor loadings to be equal on all measurement occasions) and a strong invariance model (constraining the factor loadings and the intercepts to be equal on all measurement occasions) (Little, 2013).^1^ The results show that a weak invariance model fitted the data equally well compared to the baseline model (baseline model: χ2 = 680.08, *df* = 360, *p* < 0.001, χ2/*df* = 1.89, CFI = 0.97, TLI = 0.97, RMSEA = 0.04, SRMR = 0.03; weak invariance model: χ2 = 693.33, *df* = 376, *p* < 0.001, χ2/*df* = 1.84, CFI = 0.97, TLI = 0.97, RMSEA = 0.04, SRMR = 0.04, Δχ2 = 12.51, Δ*df* = 16, ns, ΔCFI = 0.000), and the fit did also not decline for the strong invariance model (strong invariance model: χ2 = 709.19, *df* = 392, *p* < 0.001, χ2/*df* = 1.81, CFI = 0.97, TLI = 0.97, RMSEA = 0.04, SRMR = 0.04, Δχ2 = 14.92, Δ*df* = 16, ns, ΔCFI = 0.000).^2^

We investigated the longitudinal relationship between transformational leadership, fair task distribution, and emotional exhaustion, using a cross-lagged panel design in four steps. First, we specified a *stability model*, where specific variables predicted the same variables over time. Control variables were specified to be related to all constructs at all measurement occasions. This model was used as a comparison model for all subsequent models. Second, we specified a *causal effects model*, in which we investigated the proposed causal effects (transformational leadership predicting fair task distribution, and emotional exhaustion over time; fair task distribution predicting emotional exhaustion over time). Third, we specified a *reverse effects model* in which the causal ordering was reversed (emotional exhaustion predicting transformational leadership over time and fair task distribution over time; fair task distribution predicting transformational leadership over time). Fourth, we tested a *reciprocal effect* model, where causal and reverse effects were specified. These models (reversed and reciprocal) were tested to rule out alternative explanations. In line with previous recommendations regarding the test of longitudinal models, residuals of the equivalent manifest variables were allowed to correlate between measurement occasions (Little et al., 2007; Hülsheger et al., 2010; Little, 2013; Vahle-Hinz, 2016).

We tested for the indirect effect using a full-longitudinal model that investigated whether the relationship between transformational leadership T1 with emotional exhaustion T3 is mediated by fair task distribution T2. Significance of the indirect effect was tested using bias-corrected bootstrapping.

## Results

Descriptives and correlations of all study variables are displayed in Table 1. All models showed a good fit to the data (Table 2).

**TABLE 2:** Model comparisons.

**Model**	**χ^2^**	***Δ*χ*^2^***	***df***	***Δdf***	***p*_model comparison_**	**χ^2^/*df***	**CFI**	***Δ*CFI**	**TLI**	***Δ*TLI**	**RMSEA**	**RMSEA 90% CI**	**SRMR**
Stability model	1017.51		557			1.827	0.965		0.960		0.042	(0.037;0.046)	0.074
Causal effect model	944.18	68.43	551	6	0.000	1.714	0.970	−0.005	0.966	−0.006	0.039	(0.034;0.043)	0.037
Reverse effect model	983.94	31.79	551	6	0.000	1.786	0.967	−0.002	0.962	−0.002	0.041	(0.036;0.045)	0.065
Reciprocal effect model	926.21	88.46	545	12	0.000	1.699	0.971	−0.006	0.967	−0.007	0.038	(0.034;0.042)	0.036

**N* = 429–479; differences in χ^2^ between comparative models are based on Satorra and Bentler (1999) scaled chi-square difference as MLR estimator was used. Sample characteristics: organizational tenure *M* = 15.63 years (*SD* = 8.33), work hours per week *M* = 37.47 h (*SD* = 4.22 h); 4 participants (1.7%) unskilled blue collar workers, 15 (6.2%), skilled blue collar workers, 38 (15.7%) lower level white collar workers, 80 (33.1%) intermediate white collar workers, 91 (37.6%) higher level white collar workers, 7 (2.9%) were senior managers.*

**FIGURE 1:**
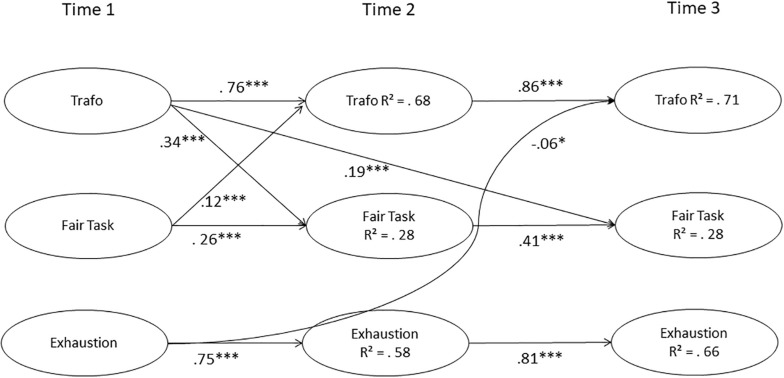
Results of the reciprocal model. Correlations not included in the figure, but latent variables were allowed to correlate within measurement occasions; control variables are not shown, but their effect is modeled at all measurement occasions; model fit: 926.21, *df* = 545, *p* < 0.001, χ^2^/*df* = 1.69, CFI = 0.97, TLI = 0.97, RMSEA = 0.04, SRMR = 0.04; ^∗^*p* < 0.05, ^∗∗∗^*p* < 0.001.

We chose the reciprocal effect model to interpret causal and reverse effects (Figure 1), as this showed overall the best fit to the data, also in comparison to the causal effect model (χ^2^(6) = 17.93, *p* < 0.01).

Transformational leadership T1 is a significant predictor of fair task distribution T2 and T3. Fair task distribution T1 is a positive predictor of transformational leadership T2 (not T3). Accordingly, the relationship between transformational leadership and fair task distribution is reciprocal over 13 months, whereas this is not true for a longer time interval (20 months). The results also show a reverse causation effect, that is, emotional exhaustion T1 is a negative predictor of transformational leadership T3. This effect seems to take considerable time to unfold, as no reverse effect of emotional exhaustion T1 on transformational leadership T2 was discovered.

The mediation analysis revealed no indirect effect of transformational leadership on emotional exhaustion via fair task distribution (χ*^2^* = 1005.45, *df* = 536, *p* < 0.001, χ*^2^/df* = 1.88, CFI = 0.96, TLI = 0.96, RMSEA = 0.04, SRMR = 0.05; (−0.002, 95% CI [−0.032, 0.029]). However, running mediation analysis with a model excluding stability effects (specifying only the necessary mediational paths for a full-longitudinal model) revealed an acceptable fit to the data (χ*^2^* = 1418.42, *df* = 527, *p* < 0.001, χ*^2^/df* = 2.69, CFI = 0.93, TLI = 0.92, RMSEA = 0.06, SRMR = 0.12), and a significant indirect effect of transformational leadership T1 on emotional exhaustion T3 via fair task distribution T2 [−0.075, 95% CI (−0.134, −0.017)]. Therefore, H1 is partially supported.

## Discussion

The present study contributes to existing research explaining the relationship between transformational leadership and follower well-being, focusing on an as-yet understudied aspect of organizational fairness. Here, transformational leadership related to fair task distribution over time. This effect was reciprocal at a shorter time lag (13 months) but appeared to be only one-directional using a longer time interval (20 months). Furthermore, excluding stability effects, fairness of task distribution mediated between transformational leadership and emotional exhaustion. These findings are important, given that the distribution of tasks lies at the core of work organization within social systems and given the relevance of coordinating team member activity to the success of these systems (Mikula, 2002). Employees are likely to have clear and socially shared ideas/norms (such as equity) about the appropriateness of given task distributions in their work teams (Mikula, 2002). Based on their experience of entitlement and their use of social comparisons, people “would be very responsive to issues of their own and other’s entitlement-deserving” (Lerner, 1987, p. 118) and to their own and relevant others’ input-output ratios. Employees judge the fairness of the task distribution depending on the extent of transformational leadership they perceive, suggesting a monitoring that is typical for exchange relationships. It is worth noting that the majority of studies was analyzing positive valued outcomes (Mikula, 2002). By focusing on task distribution, we provide one of the few studies that specifically looks at potentially negatively valued objects (work load). Furthermore, as an additional, intangible resource that can be exchanged via social relations at work, task allocation extends the number of (positively valued) resources that can be distributed according to social resource theory (Foa, 1971; Foa and Foa, 1974), originally stated as being six. On a more general level, we provide (so far limited) evidence for the mediating function of fairness perceptions between transformational leadership and employee outcomes for a specific type of fairness, namely the allocation of work tasks. In contrast to the mainly cross-sectional studies of this subject field (Skakon et al., 2010), our longitudinal study could not confirm a direct relationship between transformational leadership and emotional exhaustion. This result is in line with the longitudinal study by Gregersen et al. (2014), which could not confirm this relationship either. Interestingly, however, our results suggest a reverse causation effect over a longer time span, relating emotional exhaustion to lower transformational leadership within 20 months. This reverse effect was not previously reported, possibly indicating that emotionally exhausted employees (a) may prevent leaders from leading transformationally (e.g., hindering intellectual stimulation), or (b) perceive them to be less transformational (e.g., visions are perceived as resource-consuming instead of inspiring). However, Nielsen et al. (2018) reported a non-significant bidirectional relationship between psychological distress and leadership. Future research should replicate this phenomenon and focus on the specific explanation of this relationship. This reverse effect is also mediated via task fairness, such that employees’ perception of fairness is affected (e.g., Lang et al., 2011), or else leaders actually allocate less enjoyable tasks (e.g., more boring ones) to employees with impaired mental well-being. Even if leaders intend to be more considerate of individual team members by lightening the challenges for those with reduced capabilities, employees might perceive this as unfair. Also, leaders might lead less transformationally when employees fail to live up to their assigned tasks. This reversed mediation should also be replicated. The present study addresses the fair distribution of tasks, which is a specific, yet thus far neglected, social resource. By expanding the perception of fairness to the social comparison regarding the allocation of tasks within a team, it thus contributes to social resource theory and equity theory.

### Limitations

By applying a longitudinal design, we provide evidence for reciprocal effects when explaining the link between leadership and follower well-being (see also Van Dierendonck et al., 2004). Common method bias is of course an issue to be considered, though according to Conway and Lance (2010) “same-method observed score correlations are actually quite accurate representations of their true-score counterparts” (p. 327). Furthermore, relationships between self-report variables are not routinely upwardly biased (Conway and Lance, 2010). The anonymity of respondents should have counteracted social desirability effects. We further considered the potential threat of common method bias in our study design by temporally separating measurements (e.g., Conway and Lance, 2010). Besides, our three main constructs have little conceptual overlap, with a general judgment of leader’s behaviors, the specific question for task distribution, and an individual well-being evaluation. The scales are reliable (i.e., α between .87 and .95), and the MBI is an established scale. As our results were susceptible to common source bias, hence inflated correlations, we took some measures: As self-reports of transformational leadership may be subject to demand characteristics, we required the followers to evaluate their superior’s leadership behavior because they are immediately affected by her/his behavior. Followers also seem best suited to judge their own well-being.

From a conceptual view, it remains unclear what participants considered to be fair distributions of tasks. Besides equity and equality (Colquitt et al., 2001), ability and reciprocity may be considered relevant criteria in fair task distribution. Thus, future studies might measure the underlying fairness principle explicitly. Furthermore, unfair distributions of tasks can also be categorized with respect to quality and quantity, thus posing the question of whether the mere quantity of tasks, or the qualitative difference between different kinds of assigned tasks, are more relevant for fairness perceptions. That is, the allocation of qualitatively more challenging tasks might also be perceived as a sign of leaders’ trust in employees’ abilities and thus be judged as a way of favorably distinguishing a specific employee from her or his co-workers. Future research should seek to clarify which of these criteria is most important for the perception of task distribution fairness. Fair task distribution may also be assessed using a more objective measure, for instance, by work diaries.

### Implications

The present study preliminarily demonstrated that leaders can lower their followers’ emotional exhaustion by distributing tasks among employees in a transparent and fair way. Fair task distribution has not gained much attention in organizational fairness research (Mikula, 2002). We recommend that this topical focus be integrated into the existing social resource theory.

Furthermore, our results indicate that transformational leadership is in fact related to enhanced perceptions of fair task distribution. Thus, the causal processes linking transformational leadership, fairness of task distribution, and follower well-being would seem to merit further investigations. Transformational leaders, we suggest, may promote followers’ well-being by maintaining a high level of transparency with respect to task distribution. For their part, followers with low levels of emotional exhaustion also tend to evaluate their leaders more positively, which may constitute an alternative explanation for the results obtained. In fact, the process may be circular.

We recommend that leaders distribute tasks as fairly as possible among all team members. This may include preceding discussions about what is perceived as a “fair” norm for the team (e.g., equity/contribution principle) and the perceived need to regulate task distribution. This can both prevent social loafing and free-riding (Mikula, 2002) and also improve health in followers. Besides, leading in a transformational way may foster mutual understanding about expectations and trust in leader’s decisions. By being transparent about task allocation, leaders protect the well-being of followers by demonstrating that they recognize and will fairly compensate individual employees’ contributions to the team.

## Data Availability Statement

The datasets generated for this study are available on request to the corresponding author.

## Ethics Statement

Ethical review and approval was not required for the study on human participants in accordance with the local legislation and institutional requirements. Written informed consent for participation was not required for this study in accordance with the national legislation and the institutional requirements.

## Author Contributions

KO, TH, and TR organized the database. TV-H performed the statistical analyses. TH wrote the first draft of the manuscript. TS, KO, TV-H, TH, and TR wrote the sections of the manuscript. All authors contributed to the conception and design of the study, manuscript revision, read, and approved the submitted version.

## Conflict of Interest

TH was employed by DB Cargo AG. The remaining authors declare that the research was conducted in the absence of any commercial or financial relationships that could be construed as a potential conflict of interest.

## Footnotes

1 We could not test measurement invariance for *fairness of task distribution*, as this was measured with only one item.

2 Note that differences in χ2 between comparative models are based on Satorra and Bentler’s 1999 scaled chi-square difference because an MLR estimator was used.
